# Comparison of intraoral radiography and cone-beam computed tomography for the detection of periodontal defects: an *in vitro* study

**DOI:** 10.1186/s12903-015-0046-2

**Published:** 2015-05-28

**Authors:** Nilsun Bagis, Mehmet Eray Kolsuz, Sebnem Kursun, Kaan Orhan

**Affiliations:** Faculty of Dentistry, Department of Periodontology, Ankara University, 06560, Besevler, Ankara Turkey; Faculty of Dentistry, Department of Dentomaxillofacial Radiology, Ankara University, 06560 Ankara, Turkey; Ministry of Health, Bolu Oral and Dental Health Centre, 14000 Bolu, Turkey

**Keywords:** CBCT, Periodontal defect, Intra-oral radiography, Fenestrations, Tunnel

## Abstract

**Background:**

This study aimed to compare the diagnostic accuracy of cone-beam computed tomography (CBCT) unit with digital intraoral radiography technique for detecting periodontal defects.

**Methods:**

The study material comprised 12 dry skulls with maxilla and mandible. Artificial defects (dehiscence, tunnel, and fenestration) were created on anterior, premolar and molar teeth separately using burs. In total 14 dehiscences, 13 fenestrations, eight tunnel and 16 without periodontal defect were used in the study. These were randomly created on dry skulls. Each teeth with and without defects were images at various vertical angles using each of the following modalities: a Planmeca Promax Cone Beam CT and a Digora photostimulable phosphor plates. Specificity and sensitivity for assessing periodontal defects by each radiographic technique were calculated. Chi-square statistics were used to evaluate differences between modalities. Kappa statistics assessed the agreement between observers. Results were considered significant at P < 0.05.

**Results:**

The kappa values for inter-observer agreement between observers ranged between 0.78 and 0.96 for the CBCT, and 0.43 and 0.72 of intraoral images. The Kappa values for detecting defects on anterior teeth was the least, following premolar and molar teeth both CBCT and intraoral imaging.

**Conclusions:**

CBCT has the highest sensitivity and diagnostic accuracy for detecting various periodontal defects among the radiographic modalities examined.

## Background

Current approaches to diagnose periodontal disease include probing of gingival tissues and radiographs to evaluate osseous support. Information derived from probing the gingival tissues in association with diagnostic imaging provides guidelines for assessing the alveolar bone height and checking for the presence of bone defects [1, 2].

Today, a number of intraoral and extra-oral imaging modalities are available to assist in the examination of the periodontal patient. Commonly used two-dimensional (2D) modalities include bitewing, periapical, and panoramic radiography. These modalities are suitable because they are easily acquired, cheap and provide high-resolution images. Additionally, all of these modalities can provide important diagnostic information indeed, but none of them without limitations [3]. They are limited by overlapping anatomical structures [4, 5], difficulty in standardization [1–5], and by underestimating the size and occurrence of bone defects [6].

Studies indicated that intra-oral radiography underestimates the alveolar bone loss due to projection errors or observer errors [7–9]. There is sample research demonstrating that funnel-shaped or lingually located defects cannot be detected and that destruction of the buccal plate can be undiagnosed or undistinguished from lingual defects [5].

For this instance, three-dimensional (3D) modalities as a cone beam computed tomography (CBCT) images of periodontal bone started to use and offers a highly informative value [10]. The use of CBCT in clinical practice offers a number of potential advantages over conventional tomography, including easier image acquisition, high image accuracy, reduced artefacts, and lower effective radiation doses [11].

Research comparing the use of 3D and 2D images in artificial bone defects have shown that CBCT has a sensitivity of 80–100 % in the detection and classification of bone defects, while intraoral radiographs present a sensitivity of 63–67 %, CBCT has also shown an absence of distortion and overlapping and the dimensions it presents are compatible with the actual size [12–14]. Although, CBCT has certain advantageous regarding 3D imaging over 2D radiographies, there are still observer dependent issues on the assessment of alveolar bone and periodontal defects. Examiner interpretation errors confound data analysis and cast doubt on the validity of results esp. while evaluating the observer agreement of alveolar bone loss.

There are so far limited studies on periodontal defects and alveolar bone loss on CBCT Imaging [4, 5, 10, 14–20]. Hence, it was considered to worthwhile to compare 2D intra-oral radiographs and 3D CBCT images on detection of different types periodontal bone defects in dry skulls using CBCT imaging.

## Methods

Using retrospective data of the literature, a power analysis (Power and Precision software, Biostat, Englewood, NJ, USA) was conducted that indicated that detection of differences between 2D radiographs and 3D CBCT images could be obtained with at least 35 defects at a power of 0.8 (alpha = 0.05). Thus, this study was conducted using 12 dry skulls with maxilla and mandible and 35 artificial defects (dehiscence, tunnel [furcation defect level III], and fenestration) which were created on incisors, premolars and molar teeth separately using burs.

The skulls were obtained from different museums in our country. All skulls were dated back 10^th^ Century from different parts of country which were approved to be used for scientific study that were given by City Culture and Tourism Authorities which are connected to Anadolu Civilization Museum.

**Fig. 1:**
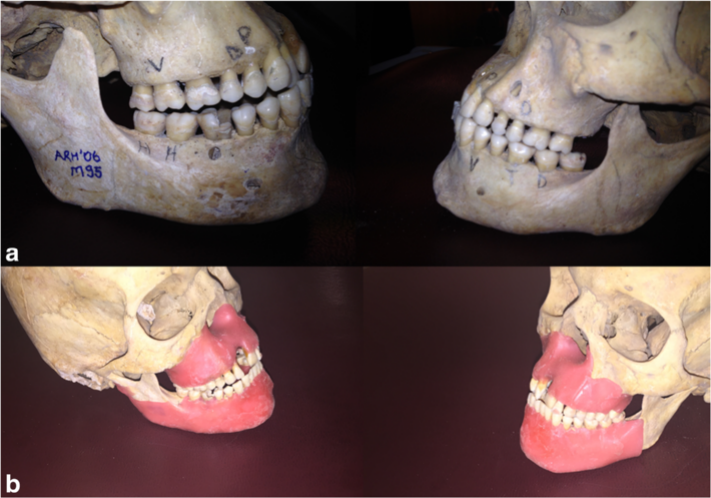
The photograph of the skulls **(a)** with defects, **(b)** and with wax covered to simulate the soft tissue

In total 14 dehiscences, 13 fenestrations, eight tunnel and 16 without periodontal defect were used in the study. These were randomly created on dry skulls. For soft tissue simulation, maxilla and mandible were covered by double layers of boxing wax (Fig. 1). The defects were created by periodontal consultant (NB) in line with Mengel et al’s study [21]. The consultant noted the periodontal defects and these were used as the Gold standard for radiographic evaluation. The periodontal defects were created using high-speed equipment with copious air/water spray and rounded diamond burs (KG Sorensen, Zenith Dental ApS, Agerskov, Denmark).

### Dehiscences

**Fig. 2:**
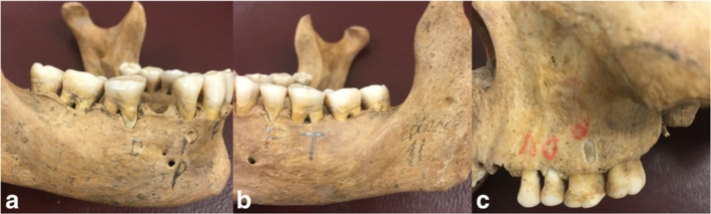
The photograph of the defects, **(a)** dehiscences, **(b)** tunnel, and **(c)** fenestration

Deshiscences were prepared in 5 molars, 4 premolars and 5 anterior teeth. The buccal bone in the coronal region of the teeth was removed until parallel walls until the walls are paralleled. The dehiscences had a standard dimension, approximately 10 mm height and 3 mm width from enamel-cement junction of the teeth (Fig. 2) 14.

### Fenestrations

Fenestrations were prepared in 5 molars, 4 premolars, 4 anterior teeth both in maxilla and mandible. The buccal bone in the central thirds of the tooth was removed until the walls ere parallel. The fenestrations had a standard dimension, approximately 4 mm height and 3 mm width (Fig. 2).

### Tunnels

All tunnel defects were prepared in mandibular molar teeth. The buccal bone lingual bone in the furcation region was removed until a continuous defect was produced. The lowest point of of the furcation was prepared as diameter of the bur, approximately 2 mm height from the furcation roof (Fig. 2).

### Radiographic imaging

**Fig. 3:**
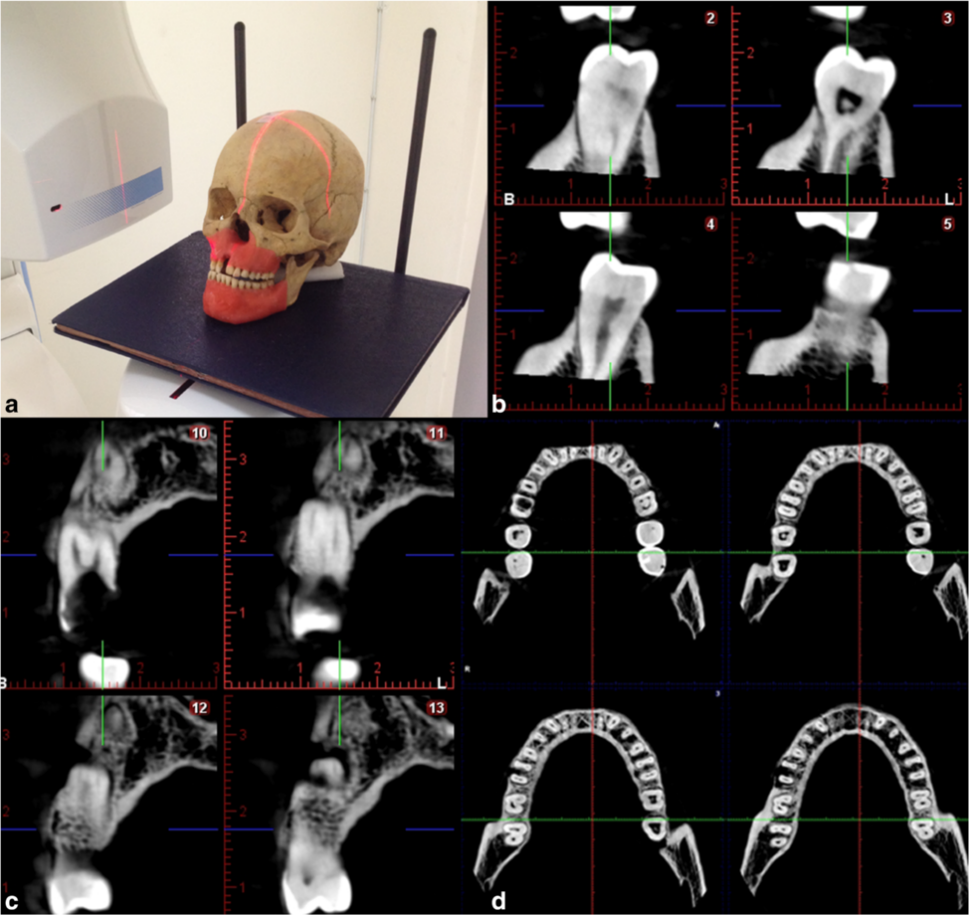
CBCT images showing **(a)** the position of the skull in the machine, **(b,c)** the periodontal defects in cross sections, **(d)** and axial planes

Each skull were exposed using a Planmeca Promax CBCT (Planmeca, Promax 3D max, Helsinki, Finland) and a Digora photostimulable phosphor plates (PSP). CBCT exposures were made in 96 kVp and 12 mA at 0.100-mm^3^ voxel size. The field of view was 5 cm in diameter and 5, 5 cm in height. Slice were 1024x1024 pixels. Axial, sagittal, cross-sectional images were reconstructed for all skulls, and 3D reconstructions were used as necessary (Fig. 3).

**Fig. 4:**
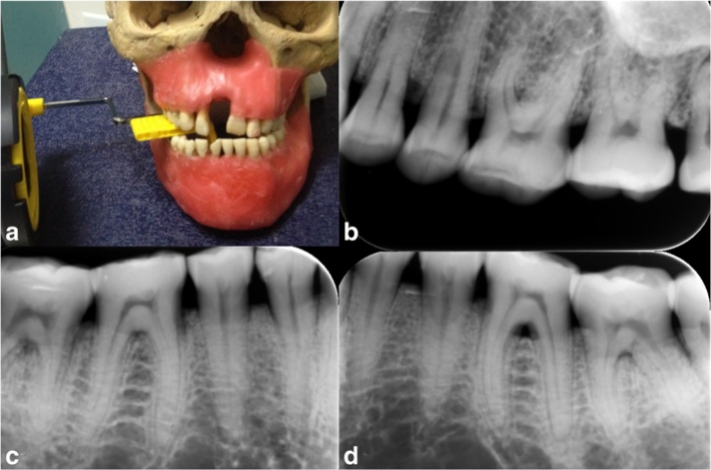
PSP intra-oral imaging **(a)** the positioning of the exposure, **(b,c,d)** the 2D images of the periodontal defect

In addition to the CBCT images, a set of digital intraoral standardized periapical images was obtained. The radiographs were obtained with an intra-oral X-ray system operating at 70 kVp, 8 mA by Evolution x3000-2c (Grugliasco, Italy) and a phosphor plate digital system (Digora Soredex, Soredex Medical Systems, Helsinki, Finland). Exposure time was 0.1 s. These were taken using parallel technique with a XCP system (Rinn Co., IL, USA) device with a 12 in. cone attached. Standardization was achieved with bite blocks that were used in all radiographic examinations. The use of the paralleling technique, complemented with a positioning holder and bite blocks, minimized image enlargement and geometric distortion of the radiographs (Fig. 4).

### Image evaluation

All digital intraoral images were saved in noncompressed file format (tagged image file format, TIFF). All images were displayed and evaluated on a 21.3-inch flat-panel color-active matrix thin-film transistor (TFT) medical display (NEC MultiSync MD215MG, Munchen, *Germany*) with a resolution of 2048 × 2560 at 75 Hz and 0.17-mm dot pitch operated at 11.9 bits. Digital intraoral images were displayed using the dedicated software of Digora imaging system (Soredex Medical Systems, Helsinki, Finland) whereas CBCT images were evaluated with its own software (Romexis 3.2, Planmeca, Helsinki, Finland). Observation conditions were optimized through use of the same computer monitor when the images were displayed. Viewing distance was kept constant to about 50 cm for the observer, and the lights were subdued during examinations.

Two dental radiologists (MEK, SK), all with 3–5 years’ experience of working with the CBCT technique examined the PSP, and CBCT images for the presence of periodontal defects in different sessions. The scores assigned by the observers were recorded by a researcher (KO) who knew the study design and had previously enhanced the images. The observers were aware that some teeth have no periodontal defects. All of the observers had access to the two views simultaneously for the intraoral and CBCT techniques. The time allocated for the observations was not restricted. Adjustment of contrast and brightness could be done, if considered necessary, using the inbuilt image display tools.

The observers were asked to define the type of the defects and also define the teeth without periodontal defects. In line with Braun’s study [10], the defects were classified being present or absent or may have been uncertain while making the diagnosis (correct, false, or questionable). In addition, all of the images were evaluated by the same examiners. For this reason, the results, positive correct and negative-correct, were summarized as “correct.” The answers: positive-false, positive-questionable, and negative-false and negative-questionable were considered “incorrect.” The level of significance was accepted at p <0.05.

All observers inter and intra evaluations were compared according to Gold standard which were created and noted by the periodontal consultant. Specificity and sensitivity for each radiographic technique were calculated. Kappa statistics was used for assessing the agreement between observers using the NCSS 2007 statistical software (NCSS and GESS, NCSS, LLC. Kaysville, UT, USA). Kappa statistics were used to determine inter and intra-observer agreement. The kappa values were interpreted according to guidelines of Landis and Koch adapted by Altman [22]. k ≤0.20 Poor, 0.21-0.40 Fair, 0.41-0.60 Moderate, 0.61-0.80 Good, 0.81-1.00 Very good. The determination of the significance level was done using the McNemar test using paired samples. Results were considered significant at p < 0.05.

## Results

Table 1 shows mean inter observer agreement for the radiographic modalities. CBCT showed a significantly greater value than the PSP. Significant difference was found between PSP and CBCT. The kappa values for inter-observer agreement between observers ranged between 0.78 and 0.96 for the CBCT, and 0.43 and 0.72 of intraoral images. The Kappa values for detecting defects on anterior teeth was least, following premolar and molar teeth both CBCT and intraoral imaging (Table 1).

**Table 1:** Inter-observer kappa coefficients among observers for first and second readings according to region

	Molar		Premolar		Anterior	
	First reading Obs1-Obs2	Second reading Obs1-Obs2	First reading Obs1-Obs2	Second reading Obs1-Obs2	First reading Obs1-Obs2	Second reading Obs1-Obs2
**PSP**	0.714	0.72	0.62	0.693	0.546	0.43
**CBCT**	0.9	0.96	0.91	0.924	0.78	0.73
**pvalue**	**p < 0.05**	**p < 0.05**	**p < 0.05**	**p < 0.05**	**p < 0.05**	**p < 0.05**

Table 2 shows the Kappa values for intraoral digital sensor and the CBCT images assessed by the two observers. Considering the observer means, cone beam dental CT images revealed significantly higher sensitivities (P < 0.05) than the intraoral systems between which no significant differences were found. The kappa values for intra-observer agreement between observers ranged between 0.42 and 0.816 for the intra-oral evaluations and 0.73 and 0.924 for the CBCT evaluations. The Kappa values for detecting defects on anterior teeth was least, following premolar and molar teeth both CBCT and intraoral imaging (Table 2).

**Table 2:** Intra-observer agreement calculated for each observer by image type according to the regions

	Molar	Premolar	Anterior	Molar	Premolar	Anterior
	Obs1 First-Second reading	Obs1 First-Second reading	Obs1 First-Second reading	Obs2 First-Second reading	Obs2 First-Second reading	Obs2 First-Second reading
**PSP**	0.706	0.816	0.811	0.693	0.546	0.42
**CBCT**	0.906	0.907	0.916	0.924	0.77	0.73

Table 3 summarizes the survey results for defect types “dehiscence”, “tunnel” and “fenestration.” Bony dehiscence, the statistical analysis showed that CBCT statistically significant better results than the conventionally used two-dimensional radiograph. Similarly CBCT again showed better performance on detecting the tunnel and fenestrations than 2D radiographs (p <0.05).

**Table 3:** Evaluation according to defect types “dehiscence”, “fenestration” and “tunnel”

	Dehiscence		Fenestration		Tunnel	
	PSP	CBCT	PSP	CBCT	PSP	CBCT
**Positive correct**	46.80 %	78.20 %	25.70 %	89.10 %	15.30 %	79.20 %
**Positive false**	41.20 %	21.80 %	62.90 %	8.14 %	82.20 %	15.40 %
**Positive-questionable**	12 %	0 %	11.40 %	2.76 %	2.50 %	5.40 %
**Negative correct**	86.40 %	93.40 %	85.20 %	95.30 %	62.90 %	75.70 %
**Negative false**	9.40 %	5.40 %	1.80 %	1.20 %	30.10 %	18.20 %
**Negative-questionable**	4.20 %	1.20 %	13 %	3.50 %	7 %	6.10 %

## Discussion

Plain conventional radiography is the most commonly used method to aid in the diagnosis of periodontal defects because of its low cost, convenience, and high resolution. However, while evaluating the images, with conventional 2D image is hard to identify a 3D structure (defects), when interpreting these radiographs esp. periodontal defects a third dimension is crucial in order to identify the nature and the course of the defects [14–20]. The present study compared the diagnostic accuracy of CBCT scans and PSP in the detection of periodontal defects. Both intra–and inter-observer agreement values for CBCT were relatively better than PSP intra-oral radiographies. The highest kappa values were obtained with CBCT images of the molars, following premolar and the anterior teeth. Overall, CBCT 8x8-cm Field of view (FOV) was found to detect periodontal defects significantly better than PSP which are in-line with previous studies [4, 5, 14–18, 20, 21, 23, 24].

Gomes-Filho et al. [23] compared the artificial induced periodontal defects with digital photographs and conventional radiographs by evaluation of three examiners. They classified the defects as; horizontal, vertical, interdental crater, one, two, three-wall infrabony defects, septum bone defect. In conclusion they stated that such diagnoses for different types of periodontal defects are extremely difficult to make. In line with our study PSP 2D images were the lowest Kappa values for detecting the periodontal defects esp. in the anterior region. Fleiner et al. also investigated the periodontal bone level using CBCT images. They conclude the CBCT would allow an accurate assessment of bone levels and description of infra-bony defects esp. %100 for crater and furcation [15]. Similarly Vandenberghe et al. [14] and Misch et al. [5] also found a %100 perfect of detection rate of periodontal defects. Our results are different than their results since we didn’t investigate the craters and infrabony defects. Similar to our study Braun et al. [10] created periodontal defects including dehiscence and fenestrations, the percentage of the correct diagnoses using three dimensional projections was very high (about 70 to 99 %). Our results are also similar to their result which were 78 % to 95 % (Table 3). Our results also confirmed that the CBCT has better diagnostic performance than PSP intra-oral images [4]. One aspect of the study that we used 0,100 mm^3^ isotropic voxels, the resolution in different machines may affect the detection of the periodontal defects which can be a further study.

Vasconcelos et al. [16] conducted a study to compare the periapical radiographs and CBCT. They conclude that the two methods differ when detecting the height of the alveolar bone crest but present similar views of the depth and width of bone defects. CBCT was the only method that allowed for an analysis of the buccal and lingual/palatal surfaces and an improved visualization of the morphology of the defect which are in line with our study results. Mengel et al. [21] also investigated the periodontal defects in CBCT. They compared the dehiscences, fenestration and furcation defects which are similar to our study. CBCT in their study found more accurate and close to histopathologic investigation of the specimen. Grimard et. al. [18] compare the direct surgical measurement with CBCT and intraoral radiographs. They found that CBCT correlated strongly with the surgical measurement whereas intra-oral radiographies correlated less favorably. Walter et al. [24] studied three dimensional CBCT images for evaluating the maxillary molar furcation involvement. According to their study the furcation involvement in clinical finding that confirmed in the CBCT in only 27 % of the sites, while 29 % were overestimated and 44 % revealed an underestimation according to CBCT analyses. The overall agreement was “moderate,” with a Cohen’s weighted k 0.518 (95 % CI: 0.269–0.767).

Umetsubo et al. [25] also evaluated CBCT imaging of early incipient periodontal defect using chemical creation of the defects. They found moderate levels of intra and inter-observer agreement for detection of the defects. The variations in Kappa values for intra–and inter-observer agreement (0.41–0.59). Our results can be different from the current study since our study was based on periodontal defects rather than incipient lesions. Moreover, our study revealed that the tunnel in molar furcation defects had values about 0.69 to 0.90 which are from good to very good agreement in detection of these defects.

This may due to different voxel size of the machines. Vanderberge et al. [20] in other study evaluated the detection of crater and furcation involvements. The figured out that 29 % of the craters and 44 % of the furcation defects were not detected and only 29 % and 20 % of the variables, respectively, were correctly classified. Our results were 41.20 % of the dehiscence, 62.90 % of the fenestrations and 82.20 % of the tunnel were positive false whereas 46.80 % of the dehiscence, 15.30 % of the tunnel and 25.70 % fenestrations were positive correct. On the CBCT images, in the same study, it was found the defects showed a 100 % detectability, while 91 % of the craters and 100 % of the furcation involvements were correctly classified. Our results on CBCT images was between 79, 20 % to 89.10 % for positive correct and 75, 70 % to 95.30 % for negative correct for CBCT images.

The quality and diagnostic accuracy of CBCT images can be significantly affected by scatter and beam hardening artifacts caused by high-density adjacent structures, such as enamel, and radio-opaque materials, such as metal posts, restorations, and root filling materials [26]. Other artifacts that may obscure radiographic findings include patient movement during the scan and volume reconstruction. In this study, we used an *in vitro* model and teeth with artificially induced periodontal defects. To prevent artifact formation on the CBCT images, no posts or metal materials were used in the root canals.

The results of this study indicated similar results with previous studies that showed better detection rates for CBCT than the digital PSP plates for detecting the periodontal defects. In our study, we divided the regions into three as; molar, premolar and the anterior. Although no statistical significance was found between the periodontal defects individually for PSP and CBCT. The diagnostic performance in the anterior region found to be the lowest in both PSP and CBCT images. This can be due to the CBCT systems used in the present study that could focus on a FOV as 5 × 5.5 cm. Therefore, we were unable to radiologically analyze indirect signs of bony lesions, which can be observed as halo lesions, perilateral radiolucency, or angular resorption of the crestal bone, combined with diffuse or defined (but not corticated) borders because of considerably larger field of view. This issue can be thought to the limitation of the study.

Several studies were used natural defects [14, 23], chemical [27] or burs [5, 10, 21] in order to create periodontal defects. The periodontal defect simulations were made using burs which can be a limitation of the study. The simulated model produced by burs, may not be the best method to evaluate the periodontal defects. Since, these created defects are well-defined structures or cavities but may not capture the natural architecture of the periodontal structures. Future studies should be conducted with chemical creation or with natural defects, even can be compared according to creation method of the defects.

Another limitation of the study can be the wax using as a soft tissue simulator in the study. Various materials simulating soft tissues used such as: water, wax, self-polymerizing resin, acryl, paraffin polyethylene and Plexiglas [28–33]. Water is to first used material in order to simulate soft tissue which was studies by Blake, et al. [30], and Borg et al. [31] also used water in order to simulate soft tissue to the specimen which was attached to jaw. Brand et al. [33] conducted a study in order to establish a phantom for radiation studies. They concluded that this type of phantom with soft tissue simulation can be used for radiology studies. Most of the previous studies esp. in intra-oral imaging concluded that wax can serve as reliable method for soft tissue simulation [20, 28, 33]. However, very limited information is available for soft tissue simulation in CBCT. Thus, again further studies should be conducted in order to evaluate the methods of soft tissue simulation in CBCT.

It is clear that CBCT is still not the first choice for periodontal bone support imaging [25]. Although the CBCT images were superior in diagnostic efficacy to conventional intraoral imaging, CBCT images should not necessarily replace intra-oral images. CBCT studies cause higher radiation exposures (4 to 20 times greater). From the standpoint of radiation risk, CBCT appears to have three to seven times the risk of a panoramic examination depending on the area examined, the degree of collimation and the acquisition software version. Thus, the decision to select an imaging modality for diagnostic purposes can be dependent for case based and should be based on the diagnostic yield expected, and in accordance with the ALARA (As Low As Reasonably Achievable) principle [34, 35].

## Conclusion

In conclusion, based on our results, CBCT has the highest sensitivity and diagnostic accuracy for detecting various periodontal defects among the radiographic modalities examined. Further studies should be taken with different FOVs and different voxel sizes of the CBCT machines. However, from the radiation protection point of view, the diagnostic information of CBCT must improve the treatment results without such a benefit this technique should not be recommended.

## Abbreviations

2D: two-dimensional
3D: Three-dimensional
CBCT: Cone beam computed tomography
PSP: Photostimulable phosphor plates
FOV: Field of view
TIFF: Tagged image file format
TFT: Flat-panel color-active matrix thin-film transistor
